# Mechanisms of exercise-induced reduction in peripheral arterial stiffness

**DOI:** 10.1007/s00421-025-05821-2

**Published:** 2025-06-28

**Authors:** Natalia S. Lima, Ronald E. Jackson, Brooks A. Hibner, Sara R. Sherman, Bo Fernhall, Tracy Baynard, Craig Crandall, Shane A. Phillips, Rrita Zejnullahi, Philip S. Clifford

**Affiliations:** 1 Integrative Physiology Laboratory, College of Applied Health Sciences, University of Illinois at Chicago, 1919 W Taylor St MC528, Chicago, IL 60612 USA; 2 Department of Exercise & Health Sciences, University of Massachusetts-Boston, Boston, MA USA; 3 Department of Internal Medicine, University of Texas Southwestern, Dallas, TX USA; 4 Department of Physical Therapy, University of Illinois at Chicago, Chicago, IL USA

**Keywords:** Pulse wave velocity, Blood flow, Compression, Handgrip

## Abstract

**Introduction:**

Peripheral arterial stiffness measured with pulse wave velocity (PWV) is reduced 5-min after an acute bout of dynamic exercise. The mechanism for the reduction in peripheral arterial stiffness is unknown. We hypothesized that increased blood flow and compression of the vasculature are potential mechanisms involved in post-exercise reduction in peripheral arterial stiffness.

**Methods:**

Brachial-radial PWV was measured with tonometers on the exercising arm of 20 healthy young volunteers (10 females, 30 ± 5 yrs, mean ± SD) before and after 5 min of rhythmic handgrip exercise at 30% of maximal voluntary contraction (MVC), 50% MVC, and a 5 min set of passive forearm compressions. Brachial blood flow was monitored with Doppler ultrasound during exercise/compression.

**Results:**

Brachial-radial PWV was lower than baseline at 5 min post (p < 0.001) for 30% MVC, 50% MVC, and compression, with responses similar among all conditions (p > 0.05). PWV remained lower than baseline at 15 min and 30 min only for 50% MVC. Blood flow and changes in brachial diameter during exercise/compression were not factors in the PWV reduction (p > 0.05).

**Conclusion:**

These findings suggest that compression of the forearm vasculature contributes to the initial reduction in peripheral PWV after handgrip exercise, but the reductions in peripheral PWV are not associated with changes in blood flow.

## Introduction

The effect of an acute bout of dynamic exercise on central and peripheral stiffness has been the target of a large body of research (Stoner et al. 2020; Naka et al. 2006). Some, but not all studies have observed reductions in central and peripheral arterial stiffness after acute exercise (Kingwell et al. 1997; Heffernan et al. 2007a). Although the findings for central arterial stiffness are inconsistent, a recent meta-analysis indicated there is better consistency in the findings of a reduction of peripheral stiffness after dynamic exercise (Saz-Lara et al. 2021). Ranadive et al. (Ranadive et al. 2012) demonstrated that dynamic exercise with either arms or legs only reduced peripheral stiffness of the limbs involved in the activity (Ranadive et al. 2012). Similarly, Sugawara showed a significant reduction in peripheral arterial stiffness in an exercised limb with no changes in the contralateral resting limb (Sugawara et al. 2003). Other studies have also shown that arterial stiffness is reduced only in the exercised limb (Siasos et al. 2016; Naka et al. 2003; Campbell et al. 2011). Together, these findings suggest that the reduction in peripheral arterial stiffness is a result of local factors. However, none of these studies examined more than a single intensity of exercise and none explored the mechanism(s) responsible for the reduction in peripheral arterial stiffness with an acute bout of exercise. Potential local mechanisms that may contribute include increases in blood flow, mechanical deformation of the vasculature caused by muscle contractions, and increases in metabolite production.

Since increases in limb blood flow are associated with reductions in arterial stiffness after heating (Cheng et al. 2021) and reactive hyperemia (Stoner et al. 2020; Naka et al. 2006; Jackson et al. 2023), we considered that post-exercise reductions in peripheral arterial stiffness may be mediated by increases in blood flow. It is well known that the magnitude of increase in blood flow during exercise is proportional to the intensity of the exercise (Bockman 1983; Armstrong and Laughlin 1985). Elevated blood flow increases conduit artery shear stress which could influence peripheral arterial stiffness via vascular smooth muscle relaxation (Pyke and Tschakovsky 2005). To our knowledge, the role of blood flow in the acute reduction of peripheral arterial stiffness after exercise is unexplored.

Another potential local factor that may contribute to the reduction of peripheral arterial stiffness is the vascular compression caused by muscle contractions. Contractions produce high intramuscular pressures sufficient to collapse arteries (Sejersted et al. 1984), a mechanical effect that can be mimicked by rhythmic cuff inflation (Roseguini et al. 2011). Vascular compression causes vasodilation (Clifford et al. 2006), increases conduit artery blood flow (Tschakovsky et al. 1996; Kirby et al. 2007), and reduces peripheral arterial stiffness (Heffernan et al. 2007b). We reasoned that comparing PWV responses following muscle contraction with passive compression could account for the mechanical effects of the contraction by creating a condition that mimics the vascular compression without accumulation of metabolites.

Therefore, the purpose of this study was to investigate the role of exercise intensity and mechanical compression in the post-exercise reduction of peripheral arterial stiffness. We hypothesized that the magnitude of reduction in peripheral stiffness is proportional to the intensity of exercise and the accompanying increase in blood flow. We also hypothesized that there would be a larger reduction in peripheral arterial stiffness after exercise than after passive compressions.

## Methods

### Ethical approval

The procedures in this study were approved by the Institutional Review Board at University of Illinois at Chicago (2022–1129) and in accordance with the Declaration of Helsinki. Participants provided verbal and written informed consents before participating in this study.

### Participants

Twenty young and healthy adults (24–39 years) volunteered for this study. Participants were excluded from the study if any of the following were present: smoking, cardiovascular, pulmonary, metabolic, neurological diseases, hypertension or hypotension, diabetes, obesity (body mass index [BMI] > 35 kg/m^2^), anti-inflammatory medication, and pregnancy. Women of childbearing age were tested for pregnancy with a urine-based test. Menstrual cycle was not controlled in this study since previous studies have shown little influence on peripheral PWV and blood flow (Augustine et al. 2018; Adkisson et al. 2010; D'Urzo et al. 2018). Based on the Paffenberger Physical Activity questionnaire all subjects were classified as physically active.

### Experimental procedures

This study consisted of a single visit to the Integrative Physiology Laboratory. Participants (n = 20) were asked to refrain from caffeine, alcohol, and exercise for at least 24-h before study visit. They were also asked to fast for at least 4-h before their test. After signing the consent and measurement of height and weight, participants rested quietly in the supine position for 10 min in a dark and temperature-controlled room before instrumentation, with the supine position maintained throughout the study visit.

### Measures

**Blood Pressure** was continuously monitored throughout the experiments via photoplethysmography (Finometer Pro, Finapres Medical System, Amsterdam, Netherlands) on the middle finger of the left hand. The pulsatile and mean blood pressure signals were sampled at 1000 Hz using PowerLab (AD instruments, Colorado Springs, CO, United States) for subsequent data analysis (LabChart).

**Brachial blood flow** from the right arm was measured using a high-resolution ultrasonography with a 5–13 MHz linear probe placed proximal to the antecubital fossa (Prosound Alpha 7, Hitachi-Aloka, Japan). Brachial artery diameter (B-mode) and flow velocity (Doppler mode) were simultaneously acquired using dual mode on the ultrasound and both were simultaneously visualized longitudinally on the screen throughout baseline, exercise/compression, and recovery. Mean blood flow velocity (Vm) was obtained with the probe positioned to maintain an insonation angle of < 60°. The video was recorded and post-processed using FMD Studio Cardiovascular Suite software (QUIPU, Pisa, Italy) at a frequency of 25 frames per second to acquire continuous measures of blood flow velocity and mean diameter during baseline, exercise or passive compressions, and recovery. Sec-by-sec data were exported to an Excel file (Microsoft, Redmond, WA) for post processing and brachial blood flow was computed using the following equation:

$${\text{BF }}\left( {{\text{mL}}\cdot{\text{min}}^{{ - {1}}} } \right)\, = \,\left( {{\text{V}}_{{\text{m}}} \, \times \,{6}0} \right){\text{ x }}(\pi r^{{2}} )$$

Where V_m_ is the mean brachial velocity in cm·sec^−1^, multiplied by 60 for conversion to cm·min^−1^ and *r*^2^ is the brachial artery radius (cm) squared. Area under the blood flow curve (AUC) was calculated with the trapezoidal method using the GraphPad Prism software (GraphPad by Dotmatics, Boston, MA).

**Brachial-radial PWV** was measured on the right arm using tonometers (Millar Instruments) held in place simultaneously on the right proximal medial brachial artery at the antecubital fossa and the longitudinal lateral radial artery near the wrist line. The locations of the tonometers were marked using a permanent marker and the average distance between tonometers was 24.2 ± 2.3 cm for all participants. The tonometer signals were sampled at 1000 Hz using PowerLab (AD instruments, Colorado Springs, CO, United States). Using a LabChart macro, PWV was calculated using the “foot-to-foot” method via the second derivative of the brachial and radial pressure waves. The transit times (foot-to-foot) of at least 7 consecutive brachial-radial waves were recorded and averaged. The distance between measurement points on the brachial and radial arteries on the right arm was measured with a tape measure. PWV was calculated as brachial-radial distance (meters) / transit time (seconds).

Pilot studies were conducted in 5 young healthy subjects to calculate the coefficient of variance (CV) in PWV from separate measurements made after repositioning the tonometers. The CV for PWV at resting baseline was 1.7% and for PWV 5-min after 50% MVC handgrip exercise was 5.0%.

### Protocol

After instrumentation, participants squeezed a handgrip dynamometer (Jamar, Bolingbrook, IL) with the right hand 3 times with maximal force, with 1 min of rest between trials. The highest value was recorded as the participant’s maximal voluntary contraction (MVC). Participants rested for at least 10 min, then performed 5 min of rhythmic handgrip exercise at either 30% or 50% of MVC in a randomized order. Exercise intensities were chosen based on pilot studies demonstrating that 50% MVC was the highest intensity that people were able to sustain for 5 min without fatigue. The exercise consisted of a 2-s contraction followed by a 2-s relaxation with visual feedback from a force gauge projected on the ceiling to achieve the right amount of force. Subjects rested for 30-min between workloads. Blood flow was measured continuously at baseline (1 min), throughout exercise (5 min) and the first 4 min of recovery (10 min total). Brachial-radial PWV was measured at baseline and 5, 15, and 30 min after contractions had ceased.

Thirty minutes after completing the second bout of handgrip exercise, participants remained in the supine position and an automated wide pressure cuff (20 cm width, model CC17, Hokanson, Bellevue, WA) was placed on their right forearm. The pressure cuff was set to rapidly inflate to 200 mmHg and deflate for 5 min (2 s on, 2 s off) using an automated cuff inflation unit (Hokanson E20, Bellevue, WA). Brachial blood flow was measured continuously at baseline (1 min), during compressions (5 min), and the first 4 min of recovery (10 min total). Brachial-radial PWV was measured at baseline and 5, 15, and 30 min after passive mechanical compressions had ceased.

### Statistical analyses

Sample size for this study was calculated based on the variability in studies from Sugawara et al. (Sugawara et al. 2003) and Heffernan et al. (Heffernan et al. 2007b). The results of the power analysis for a two-way repeated measures ANOVA interaction indicated that 18 participants were necessary to detect a meaningful difference of 0.85 m/sec in PWV with 80% power and an alpha at 0.05.

Prior to analyses, normality of the data was confirmed by using the Kolmogorov–Smirnov Test (SPSS V24, IBM Armonk, NY). PWV, blood flow, brachial diameter and blood pressure were analyzed with a two-way (condition x time) repeated measures ANOVA (using SPSS). Blood flow AUC was analyzed with a one-way repeated measures ANOVA. Post-hoc comparisons were done with a Bonferroni correction for multiple comparisons. The relative influence of multiple factors on changes in peripheral PWV was assessed with a mixed linear model (using the lmer function from the lme4 package in R statistical software, version 4.2.2) with fixed effects of condition (30% intensity and 50% intensity), time, BF AUC, brachial diameter, and baseline PWV and a random effect factor of subject ID with a random intercept nested within the subject. The variance–covariance structure for the random effects had a single intercept for each subject and no correlation between random effects (i.e., the identity matrix was used for the variance–covariance structure).

Significance level was set at α = 0.05 and all data are presented as mean ± standard deviation (SD).

## Results

A total of 20 participants was included in this study (10 females, 10 males) with their descriptive characteristics presented in Table 1.

**Table 1:** Participant characteristics

	n = 20	Females (n = 10)	Males (n = 10)
Age (years)	30 ± 5	30 ± 5	30 ± 5
Weight (kg)	76 ± 18	66 ± 12	86 ± 18
Height (cm)	171 ± 10	167 ± 11	176 ± 7
BMI (kg/m^2^)	26 ± 4	24 ± 3	27 ± 4
MVC (kg)	29 ± 9	24 ± 7	35 ± 8

*BMI* body mass index, *MVC* maximal voluntary contraction. Mean ± SD

**Fig. 1:**
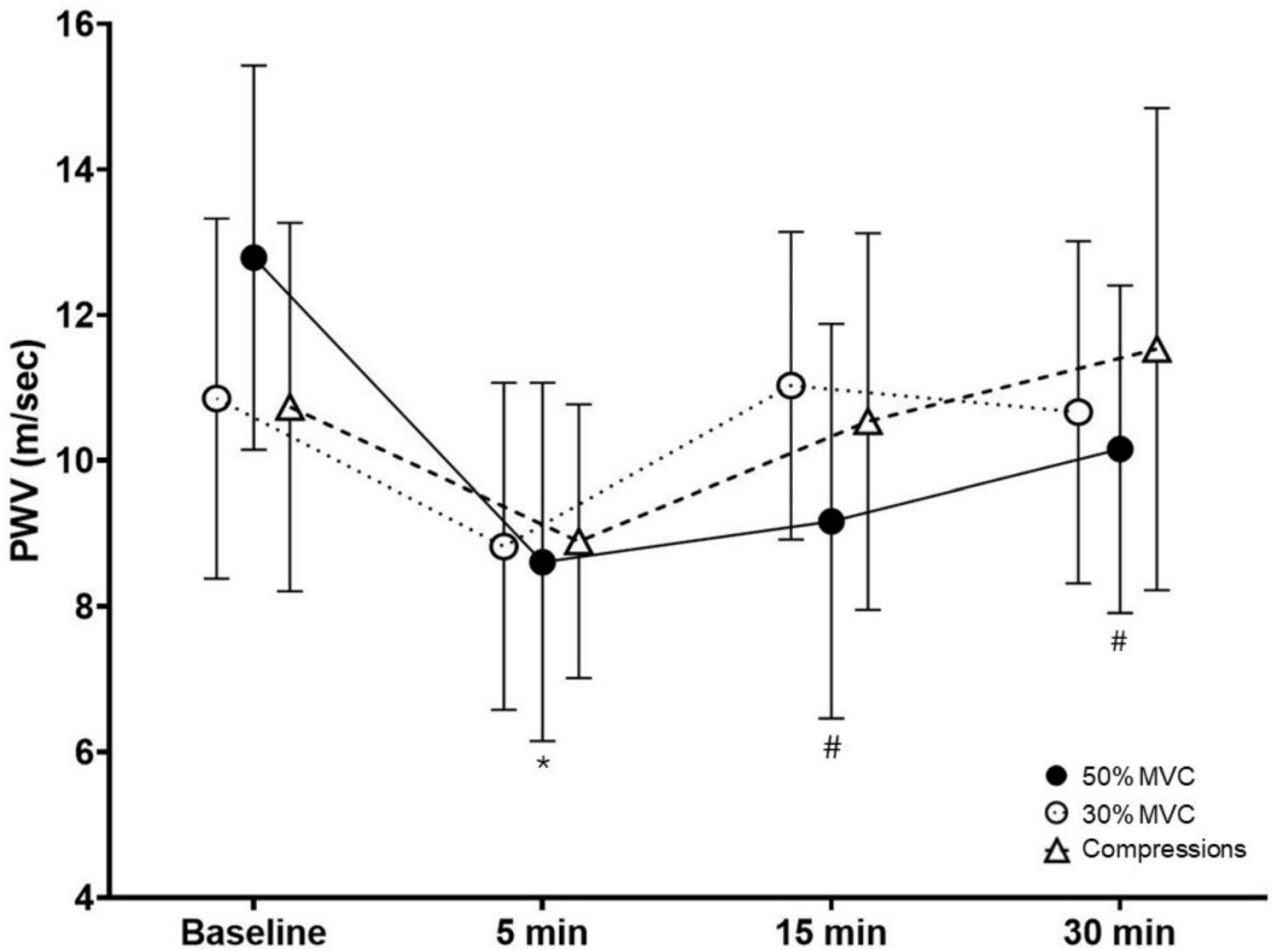
Measurements of brachial-radial pulse wave velocity (PWV) before and after 5-min of rhythmic handgrip exercise and passive mechanical compressions (mean ± SD) in all participants (n = 20). *P < 0.001 compared to baseline for all conditions. # P < 0.01 compared to baseline for 50% MVC

Figure 1 shows PWV at baseline and 5-, 15-, and 30-min after rhythmic handgrip exercise at 30% MVC, rhythmic handgrip exercise at 50% MVC, and passive compression. There was a significant time effect (p < 0.001) with PWV lower than baseline at 5-min post for all 3 conditions. There was also a significant condition x time interaction (p < 0.001) with a partial eta squared value of 0.762. Post hoc analyses showed no differences among 30% MVC, 50% MVC, and passive compression at 5 min post (all p > 0.05). Post hoc analyses also showed that 50% MVC remained below baseline at 15-min (p < 0.001) and 30-min post (p < 0.001), whereas handgrip exercise at 30% MVC and compression were not different than baseline at 15-min and 30-min post (all p > 0.05).

**Fig. 2:**
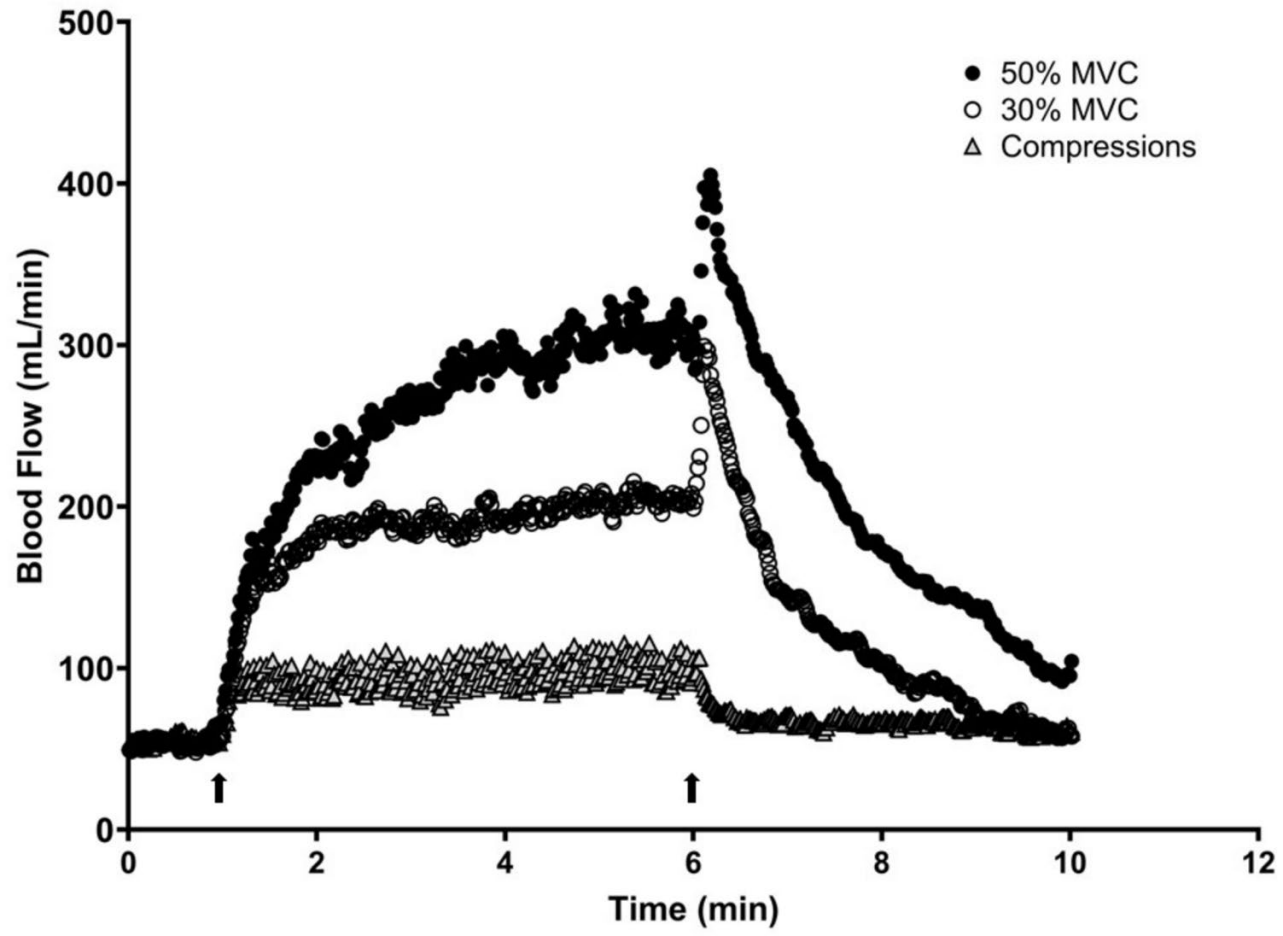
Continuous sec-by-sec brachial blood flow tracings from all participants from baseline, 5-min intervention, and 4-min recovery (10-min total). The left arrow indicates the onset of either rhythmic handgrip exercise or passive mechanical compressions of the forearm vasculature, while the right arrow indicates the end of exercise or compressions. MVC, maximal voluntary contraction

Ensemble averages of blood flow curves for all subjects during exercise and passive mechanical compressions of the forearm are shown in Fig. 2. There was a rapid increase in blood flow for both exercise intensities. As exercise progressed to steady state, blood flow increased proportionally to the intensity and remained elevated during the remainder of the exercise bout. After cessation of muscle contractions, blood flow decreased rapidly and returned to near baseline values. A similar pattern was observed with passive mechanical compressions, but the plateau occurred at a lower blood flow.

**Fig. 3:**
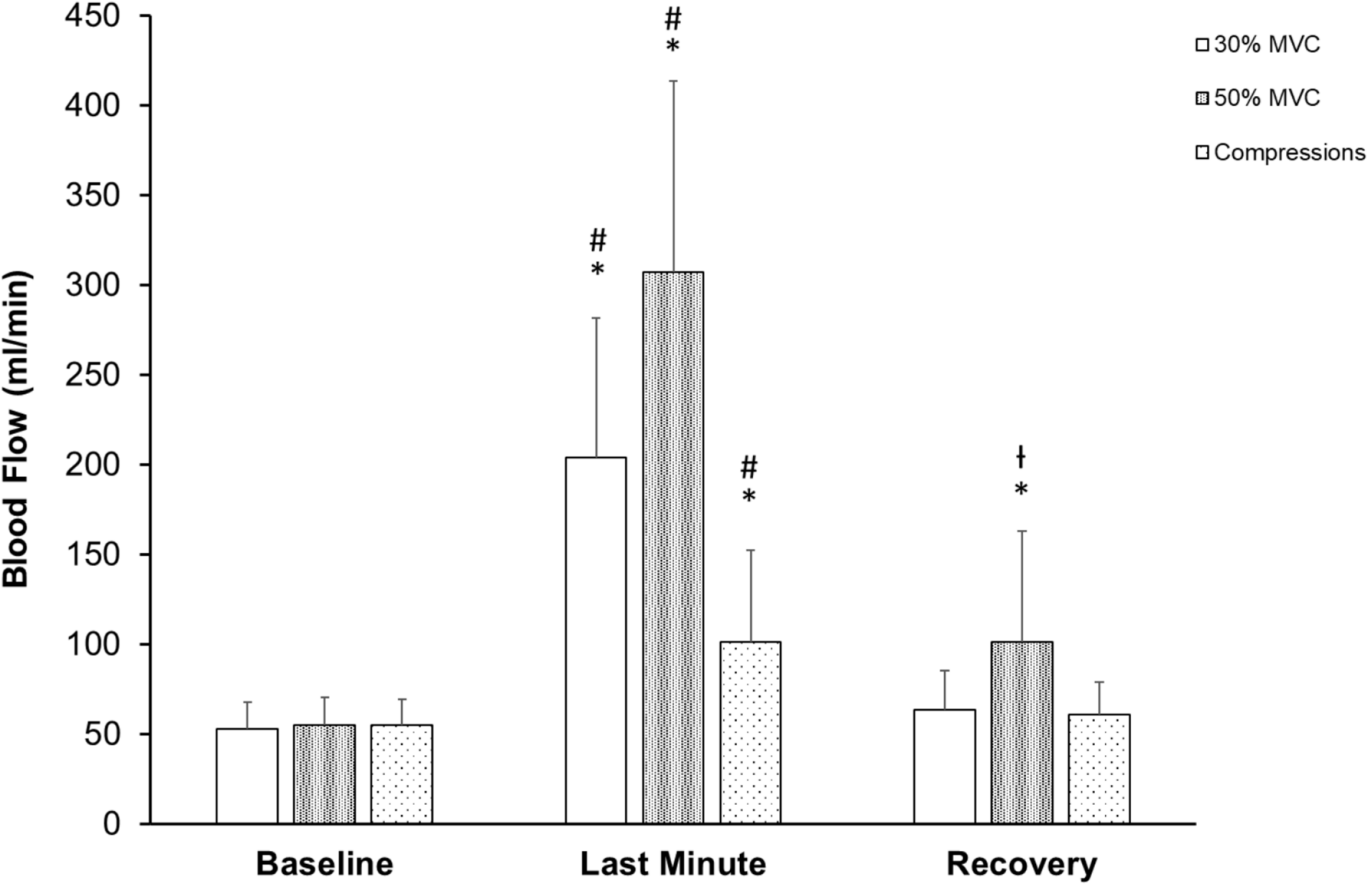
Brachial blood flow average for 1-min at baseline, the last minute of intervention (rhythmic handgrip exercise or passive mechanical compressions), and the last minute of recovery for all participants (n = 20). Data are presented as mean ± SD. * P < 0.001 compared to baseline for that intervention. # P < 0.001 compared to both other interventions. ^┼^P < 0.05 compared to both other interventions

Figure 3 displays 1-min averages of blood flow at baseline, last minute of exercise or passive mechanical compression, and the last minute of recovery. Baseline blood flow was similar among conditions (p > 0.05). There was a significant time effect (p < 0.001) with blood flow being higher during the last minute for all 3 conditions. There was also a significant condition x time interaction (p < 0.001). Post hoc analyses showed that blood flow over the last minute of each intervention was highest for 50% MVC, intermediate for 30% MVC, and lowest for compressions. Blood flow returned to baseline levels during recovery except for 50% MVC (p < 0.001).

**Fig. 4:**
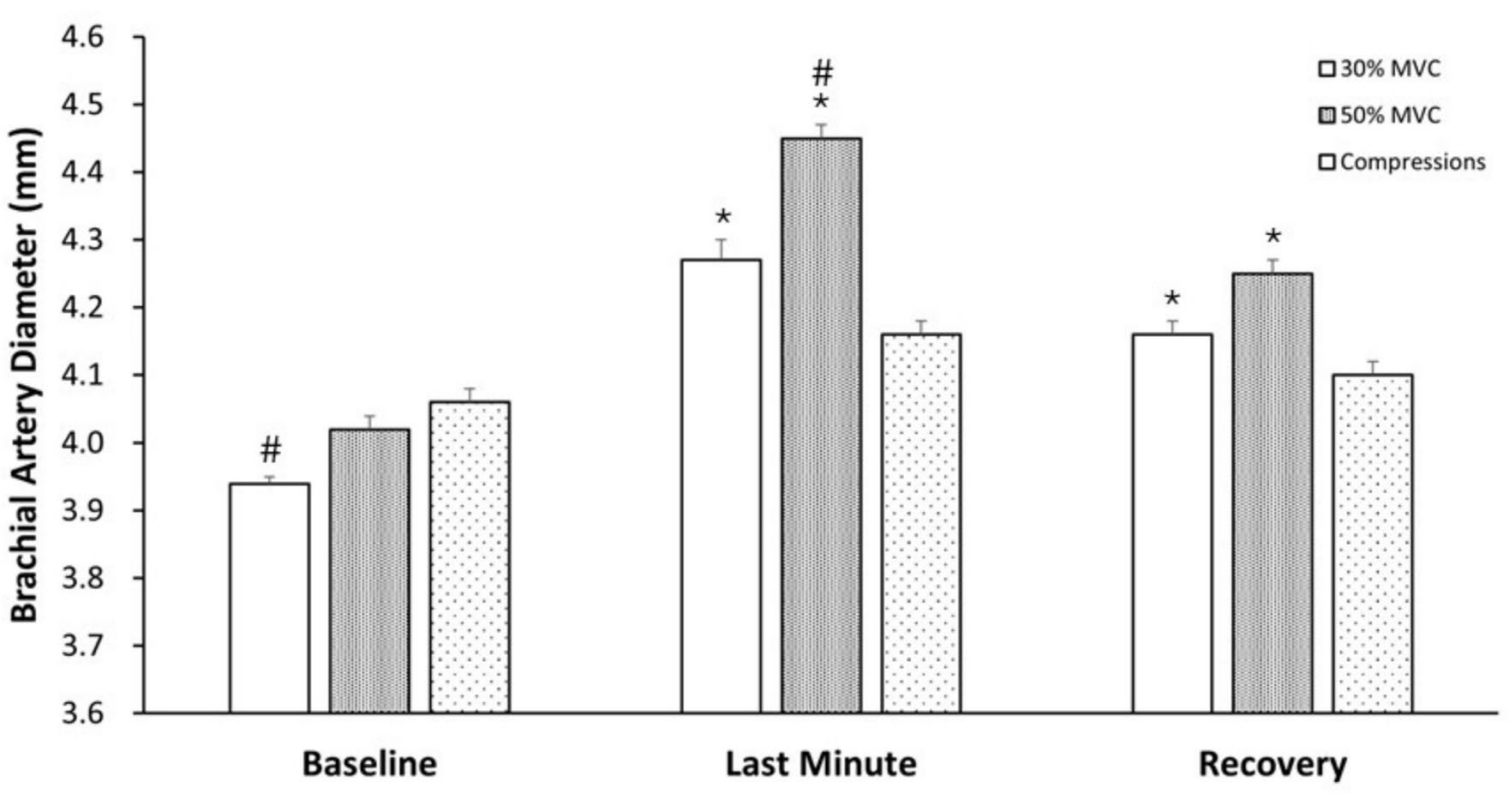
Mean brachial artery diameter for 1-min at baseline, the last minute of intervention (rhythmic handgrip exercise or passive mechanical compressions), and the last minute of recovery for all participants (n = 20). Data are presented as mean ± SD. * P < 0.001 compared to baseline for that intervention. # P < 0.001 compared to compressions for that time

Brachial artery diameters at baseline, last minute of exercise or passive mechanical compression, and the last minute of recovery followed the same general pattern as blood flow (Fig. 4). Baseline diameters were similar for 50%MVC and compression, but lower (p < 0.001) for 30% MVC compared to compression. There was a significant time effect (p < 0.001) with diameter being higher during the last minute for 30% MVC and 50% MVC, but not compressions. There was also a significant condition x time interaction (p < 0.001). Post hoc analyses showed that diameter over the last minute of each intervention was highest for 50% MVC, intermediate for 30% MVC, and lowest for compressions. At recovery, diameter remained elevated relative to baseline for 30% MVC (p = 0.003) and 50% MVC (p < 0.001).

The increase in volume of blood due to rhythmic handgrip exercise or passive mechanical compressions was calculated as the area under the curve (AUC) of the brachial blood flow from start to end of contractions/compressions. There was a significant condition effect for blood flow AUC (p < 0.001). The blood flow AUC for 50% MVC (112,954 ± 55,455 ml) was higher than 30% MVC (76,157 ± 43,476 ml, p < 0.001), and passive compression (39,935 ± 21,694 ml, p < 0.001). Blood flow AUC for 30% MVC was also higher than passive compression (p < 0.001).

The influence of selected factors (condition, time, blood flow AUC, brachial diameter, and baseline PWV) on the post-exercise related reduction in peripheral PWV at 5-min was explored using a mixed linear model. Statistically significant factors were exercise intensity, time, and baseline PWV. Passive compression relative to the reference exercise at 30% MVC, blood flow AUC, and change in brachial diameter were not statistically significant factors (See Table 2).

**Table 2:** Mixed linear model for peripheral PWV

	β_0_	β	P-value	R^2^m	R^2^c
PWV					
Exercise Intensity	2.983 (1.130)	− 1.973 (0.5968)	0.0018	0.38	0.52
Compression		0.3087 (0.6002)	0.6094		
Post 15 min		1.5470 (0.3786)	0.0001		
Post 30 min		2.1280 (0.3786)	< 0.0000		
BF AUC		0.00005 (0.000007)	0.4871		
Diameter		− 0.5814 (0.7236)	0.4256		
Baseline PWV		0.5276 (0.0784)	< 0.0000		

Mean (SE). *PWV* pulse wave velocity, *BF AUC* blood flow area under the curve. Reference for exercise intensity and compression = 30% MVC, reference for time = post 5-min. β_0_, intercept; β, regression coefficient**;** R^2^m, variance explained by the fixed effects within the model; R^2^c, variance explained by fixed and random effects

Table 3 displays the mean arterial pressure at baseline, 5-, 15-, and 30-min post-exercise and compression across all conditions. Likewise, Table 4 displays the pulse pressure at baseline, 5-, 15-, and 30-min post-exercise and compression across all conditions. There was no change in mean arterial pressure or pulse pressure at these time points (p > 0.05) which correspond to the times when PWV was measured.

**Table 3:** Mean arterial pressure (mmHg) before and after exercise or passive compression. No significant differences were observed between the 3 conditions or across time points

	30% MVC	50% MVC	Passive Compressions
Baseline	86 ± 9	88 ± 7	91 ± 7
5-min post	88 ± 10	89 ± 9	92 ± 8
15-min post	88 ± 9	87 ± 7	93 ± 8
30-min post	88 ± 8	89 ± 7	91 ± 7

Mean ± SD

**Table 4:** Pulse pressure (mmHg) before and after exercise or passive compression. No significant differences were observed between the 3 conditions or across time points

	30% MVC	50% MVC	Passive Compressions
Baseline	54 ± 9	53 ± 13	54 ± 9
5-min post	55 ± 10	53 ± 15	55 ± 11
15-min post	54 ± 10	55 ± 8	56 ± 10
30-min post	52 ± 13	54 ± 7	56 ± 9

Mean ± SD

## Discussion

The aim of this study was to examine the contributions of exercise intensity and passive mechanical compression to the post-exercise reduction in peripheral arterial stiffness measured with brachial-radial PWV. The salient findings are: 1) peripheral PWV was reduced 5-min after both rhythmic handgrip exercise and passive compression; 2) PWVs were similar 5-min after 50% MVC, 30% MVC and passive compression; 3) the reduction in PWV was sustained through 30-min for 50% MVC, whereas PWV returned to baseline levels at 15 min and 30 min post for 30% MVC and compression; 4) blood flow AUC was not significantly related with the reduction in PWV and 5) change in brachial diameter was not significantly related with the reduction in PWV. These findings suggest that compression of the forearm vasculature contributes to the initial post-exercise reduction in peripheral PWV. Contrary to our hypothesis, the reductions in peripheral PWV were not associated with the blood flow AUC.

A reduction in central and peripheral arterial stiffness after an acute bout of dynamic exercise has previously been observed (Kobayashi et al. 2017; Heffernan et al. 2007c; Kingwell et al. 1997), but the acute decrease in central arterial stiffness is not a universal finding. For example, Rakobowchuck et al. (Rakobowchuk et al. 2009) observed an increase in central arterial stiffness measured 20-min after either a single sprint interval or after multiple sprint interval sessions. More recently, another study observed that central arterial stiffness was elevated 30-min after 10-min of moderate intensity walking on a treadmill (Perdomo et al. 2019). Although there is considerable variability of responses in central arterial stiffness after an acute bout of dynamic exercise, this is not true for peripheral arterial stiffness.

Reduction of peripheral arterial stiffness with dynamic exercise is a consistent finding across multiple studies using different exercise intensities and durations (Kobayashi et al. 2017; Peres et al. 2018; Campbell et al. 2011; Ranadive et al. 2012; Naka et al. 2003; Kingwell et al. 1997). However, no prior study has explored the effect of graded intensities of exercise on peripheral arterial stiffness. We measured PWV 5-min after rhythmic handgrip exercise at 30% and 50% MVC. In contrast to our hypothesis, PWVs were similar 5-min after 30% MVC and 50% MVC. On the other hand, the reduction in PWV persisted through 30-min only for 50% MVC. Thus, there was an intensity effect for the duration of response but not the magnitude of the response.

There is evidence that an acute exercise-related reduction in peripheral arterial stiffness is a local phenomenon (Peres et al. 2018; Rakobowchuk et al. 2009; Tordi et al. 2010; Sugawara et al. 2003; Ranadive et al. 2012). This interpretation was particularly evident in the study by Sugawara et al. who had their participants exercise only one leg while the other leg remained inactive (Sugawara et al. 2003). Peripheral arterial stiffness was reduced in the exercised limb, but not in the inactive limb. The lack of change in peripheral arterial stiffness in the inactive leg suggests that local factors present with muscle contractions are required for a reduction in arterial stiffness This concept is supported by the results of Ranadive et al. who measured peripheral arterial stiffness after acute bouts of arm or leg exercise (Ranadive et al. 2012). Peripheral arterial stiffness was only reduced in the arterial segment of the exercised limb, while no changes were observed in the limb that remained inactive or in central PWV. Local factors that may influence the reduction in peripheral arterial stiffness after an acute bout of rhythmic exercise include mechanical compression of the vasculature by muscle contractions, metabolite production, and increased blood flow.

Muscle contractions during exercise can raise intramuscular pressure above the arterial pressure and cause compression of the vasculature (Sejersted et al. 1984). These mechanical effects can be mimicked by passive mechanical compressions (Roseguini et al. 2011). Heffernan et al. (Heffernan et al. 2007b) performed a set of rhythmic passive mechanical compressions in one leg of their participants. Passive mechanical compressions reduced peripheral PWV measured 10-min after the intervention, while no change in PWV was observed in the control limb (Heffernan et al. 2007b). Since no direct comparison was made with muscle contractions in that study, we compared rhythmic handgrip exercise with passive compression in the same individuals. The data confirm that rhythmic passive mechanical compressions of the forearm vasculature reduce peripheral PWV. Furthermore, our study extended that finding by demonstrating that passive compression and rhythmic handgrip exercise produced similar reductions in peripheral PWV. These findings are consistent with the idea that mechanical compression plays a key role in the post-exercise reduction in peripheral PWV. Further research is needed to investigate the physiological mechanisms whereby compression affects arterial stiffness. Two possibilities are pressure-induced activation of mechanosensitive ion channels (Drummond et al. 2004) or rapid depolymerization of the actin cytoskeleton (Clifford et al. 2018) in vascular smooth muscle.

One difference between the stimuli produced by passive compression and active contractions is the production of metabolites by skeletal muscle during rhythmic handgrip exercise. It is tempting to speculate that the prolonged reduction in PWV at 50% MVC could be related to metabolite release during muscle contractions. Local production of metabolites has been previously suggested as a potential factor responsible for the observed reduction in peripheral arterial stiffness with dynamic exercise (Peres et al. 2018; Rakobowchuk et al. 2009; Sugawara et al. 2003; Naka et al. 2003). However, little explanation was provided by these authors regarding how metabolites would affect peripheral arterial stiffness.

It is known that metabolites produced by exercising muscles act on the vascular smooth muscle of resistance arterioles to cause a decrease in downstream resistance which increases limb blood flow. The increase in conduit artery blood flow increases shear stress, releasing nitric oxide (NO), prostaglandins, and endothelium derived hyperpolarizing factor (EDHF) which relax vascular smooth muscle in the conduit artery. It is possible that either metabolites or flow-mediated dilation are responsible for the increase in brachial diameter observed in this study. However, the results of the mixed linear model show that neither blood flow AUC nor change in brachial diameter were related to the reduction of peripheral PWV. Although increases in limb blood flow are associated with reductions in peripheral PWV after heating (Cheng et al. 2021) and reactive hyperemia (Stoner et al. 2020; Naka et al. 2006), that does not appear to be the case for rhythmic handgrip exercise at the observed intensities. Other investigators have proposed that flow mediated dilation is essential for the changes in arterial stiffness following a reactive hyperemia stimulus (Cauwenberghs et al. 2018; Ellins et al. 2016). That also does not appear to the case for rhythmic handgrip exercise. Based on the results of the mixed linear model, we conclude that the increases in brachial blood flow and diameter did not contribute to the observed early post-exercise reductions in peripheral PWV. A caveat to this conclusion is the theoretical possibility that only small increases in conduit artery blood flow or diameter are necessary to reduce arterial stiffness and that a ceiling effect explains the similarity of the PWV responses to compression and both exercise intensities despite observed differences in blood flow and diameter. Additional experiments would be required to explore this possibility.

To our knowledge, this is the first study to measure changes in peripheral PWV and conduit artery blood flow related to rhythmic exercise and passive compressions. There are several limitations to consider. First, we did not measure the intramuscular pressure during contractions or external compressions and it is uncertain if this stimulus matched the intramuscular pressure caused by muscle contractions at 30% or 50% MVC. Rhythmic passive compressions were performed at 200 mmHg in the present study which is on the plateau of the dose/response curve shown by Kirby et al. (Kirby et al. 2007) where repeated passive compressions of 100 and 200 mmHg resulted in identical increases in blood flow. Second, based on measurements in the non-exercising arm, blood pressure was unchanged across conditions. Although blood pressure was not measured in the exercising arm where peripheral PWV was measured, recordings were made 5-min after exercise when blood pressure should have been similar in both limbs. Third, since there was a large variability in baseline PWV, a mixed linear model was performed with baseline PWV as a fixed factor to account for the variability. The results indicate that starting baseline influenced the reduction in peripheral PWV. Fourth, it is possible that an order effect influenced the results since the two exercise intensities were randomized but the order of exercise and compression was not randomized. Fifth, the MVC measurements seem low for young and healthy subjects. This finding could be due to the type of handgrip dynamometer employed though it was rigorously calibrated. It could also be related to the fact that involvement of the upper arm and shoulder muscles was scrupulously restricted. Lastly, although this study was not powered to detect significant differences between sexes, a sub-analysis of the present data indicated no statistically significant differences in PWV post handgrip exercise between men and women (p > 0.05, data not shown).

In summary, the results of this study demonstrated that peripheral PWV was reduced similarly 5-min after both rhythmic handgrip exercise and passive compression and that these reductions in PWV were not related to the accompanying increases in blood flow and diameter. These findings suggest that mechanical compression makes an important contribution to the initial reduction in peripheral PWV after exercise.

## Funding.

None to declare.

## Conflict of interest

None to declare for any authors.

## Data Availability

Data supporting the results of the present study are available from the corresponding author upon reasonable request.
